# Characterization of Shiga Toxigenic *Escherichia coli* O157 and Non-O157 Isolates from Ruminant Feces in Malaysia

**DOI:** 10.1155/2015/382403

**Published:** 2015-10-11

**Authors:** Asanthi Perera, Charles M. Clarke, Gary A. Dykes, Narelle Fegan

**Affiliations:** ^1^School of Science, Monash University, Jalan Lagoon Selatan, 46150 Bandar Sunway, Selangor, Malaysia; ^2^CSIRO, Food and Nutrition Flagship, 671 Sneydes Road, Werribee, VIC 3030, Australia

## Abstract

Shiga toxigenic *Escherichia coli* (STEC) O157 and several other serogroups of non-O157 STEC are causative agents of severe disease in humans world-wide. The present study was conducted to characterize STEC O157 and non-O157 serogroups O26, O103, O111, O121, O45, and O145 in ruminants in Malaysia. A total of 136 ruminant feces samples were collected from 6 different farms in Peninsular Malaysia. Immunomagnetic beads were used to isolate *E. coli* O157 and non-O157 serogroups, while PCR was used for the detection and subtyping of STEC isolates. STEC O157:H7 was isolated from 6 (4%) feces samples and all isolates obtained carried *stx*_2c_,  *eaeA-γ*1, and *ehxA*. Non-O157 STEC was isolated from 2 (1.5%) feces samples with one isolate carrying *stx*_1a_, *stx*_2a_, *stx*_2c_, and *ehxA* and the other carrying *stx*_1a_ alone. The presence of STEC O157 and non-O157 in a small percentage of ruminants in this study together with their virulence characteristics suggests that they may have limited impact on public health.

## 1. Introduction

Shiga toxin producing* E. coli* (STEC), a serologically diverse group of zoonotic pathogens, have emerged as one of the most virulent groups of bacteria associated with cases of food borne disease in humans [1]. STEC can cause a spectrum of diseases ranging from mild diarrhea to severe bloody diarrhea, called hemorrhagic colitis (HC), and even life-threatening sequelae such as hemolytic uremic syndrome (HUS). Patients with HUS were often diagnosed as having thrombotic thrombocytopenic purpura (TTP), although thrombotic microangiopathy is now considered a more accurate description of the condition associated with HUS caused by STEC [2]. Production of Shiga toxin (Stx) is considered as the major virulence factor of STEC [1] which contributes to the development of HUS in humans [2]. Stx production alone is not sufficient for STEC to cause disease. Accessory virulence factors include a 34 kb chromosomal pathogenicity island called the “locus for enterocyte effacement” (LEE) carrying several virulence associated genes, such as the attaching and effacing (*eaeA*) gene, and a large plasmid (60 MDa) with an* ehxA* gene encoding an enterohemolysin.* EaeA* encodes an outer-membrane protein called intimin which enables the intimate adherence of STEC to the intestinal epithelium of the host [3]. The enterohemolysin protein is implicated in extracting iron from the blood released into the intestine [4].

The prototype STEC serotype is* E. coli* O157:H7 and its ability to cause HC and HUS in many regions and countries is well established. The pathogenic potential and public health significance of several non-O157 STEC serogroups, particularly O26, O103, O111, O121, O145, and O45 referred to as the “big 6” non-O157 STEC serogroups [5], have also been described in recent years due to their association with clinical HC and HUS in humans. In some geographical areas, such as in Europe, the disease caused by non-O157 strains is significantly more common than that caused by O157:H7 [6, 7].

Ruminants are considered an important source of both* E. coli* O157 and non-O157 with cattle being identified as the primary reservoir. Intestinal carriage of* E. coli* O157 and non-O157 in ruminants results in their fecal shedding and release into the environment. As a result, infections of* E. coli* O157 and non-O157 can be transmitted to humans via the consumption of food and water contaminated by animal feces.

Data on* E. coli* O157 and non-O157 serotypes in ruminants is limited in countries of the tropical regions including Malaysia. In addition, the data reported so far on* E. coli* O157 and non-O157 in ruminants from tropical countries other than Malaysia demonstrates substantial variation in their prevalence and virulence properties. In West Bengal, India, a total of fourteen STEC O157 isolates were obtained from two (2.04%) slaughtered cattle feces samples and six (7.59%) diarrhoeic calf feces samples [8]. The majority of STEC O157 isolates (85.71%) obtained from this study carried* stx*_2_ alone. STEC O157 was obtained from 0.6% of cattle feces samples in Brazil [9], where the majority of isolates carried* ehxA* either with both* stx*_1_ and* stx*_2_ or with* stx*_2_ alone. The prevalence of* E. coli* O157 was found to be 1.25% in cattle farms in central Mexico [10]. Non-O157 STEC was found in 18% of cattle feces samples in Calcutta, India [11], in which* stx*_1_ predominated. In another study in Brazil, non-O157 STEC was isolated from 5.81% of calf feces samples [12] where* stx*_1_ was the dominant* stx* genotype observed.

Only three studies which isolated STEC O157 from beef samples have to our knowledge been conducted in Malaysia [13–15]. Apart from a single study which reported sporadic cases of STEC O157 infection among 14% of patients presented with bloody diarrhea at a local hospital in Kuala Lumpur, Malaysia [16], there are no other published reports of sporadic cases or outbreaks of STEC O157 and non-O157 in the country. Although studies have demonstrated the presence of STEC O157 in foods of animal origin, the presence and characterization of STEC O157 or non-O157 in ruminant feces from Malaysia has not yet been determined.

The aim of the present study was to examine ruminant feces samples for the presence of STEC O157 and the “big 6” non-O157 STEC serogroups in Malaysia. The isolated strains of* E. coli* O157 and non-O157 were further characterized to determine their genetic diversity and presence of virulence factors to indicate the risk potential of these strains to public health.

## 2. Materials and Methods

### 2.1. Sample Collection and Preparation

**Figure 1:**
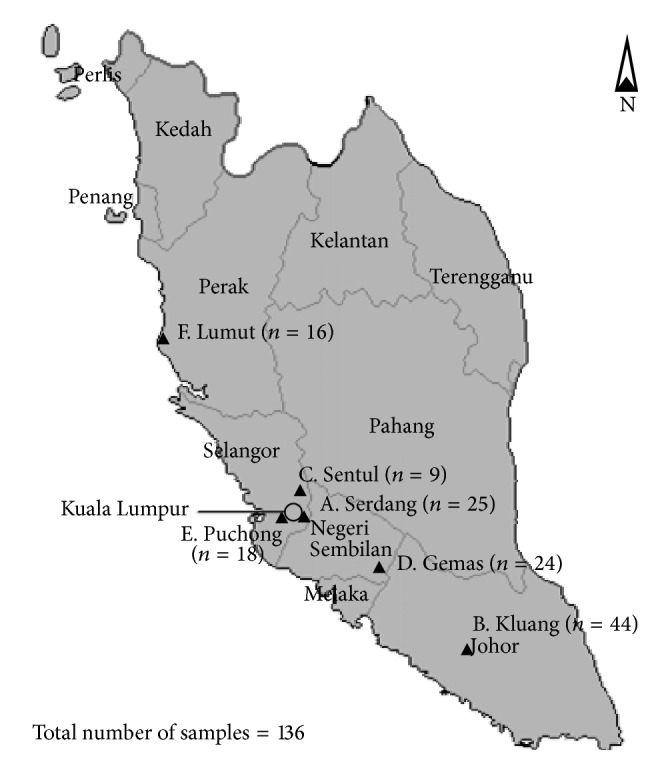
Geographical distribution of farms A–F in Peninsular Malaysia from which the ruminant feces samples were collected.

Samples were collected from six different ruminant farms in Peninsular Malaysia (Table 1). The geographical distribution of the six farms is depicted in Figure 1. Farms A, C, and F were small dairy cattle farms, while farm E was a small dairy farm consisting of cattle and goats. Farm B was also a dairy farm but with a larger number and diversity of ruminants consisting of cattle, buffaloes, goats, and sheep. Farm D was a large beef cattle farm. A total of 136 fresh ruminant feces samples (~25 g each) from cattle, buffalo, sheep, and goat were collected from the pen floors (over a period of six months) into sterile containers and were stored at 4°C on ice until processed in the lab on the same day. All fecal samples collected were divided into two 10 g samples. One of the 10 g samples was used for enrichment and the other was used for long term storage in tryptone soy broth (TSB; Merck, Darmstadt, Germany) with 25% glycerol at −70°C.

### 2.2. Isolation and Characterization of* E. coli* O157

Each fecal sample (10 g) was diluted 1/10 in buffered peptone water (BPW; Oxoid, Hampshire, UK) and homogenized for 30 s. Samples were incubated for 18 h at 37°C without agitation. Immunomagnetic separation (IMS) was performed using Dynabeads anti-*E. coli* O157 (Dynal, Oslo, Norway) according to the manufacturer's instructions. Resulting bead-bacteria complexes were spread on to sorbitol-MacConkey agar (SMAC; Oxoid, Hampshire, UK) and sorbitol-MacConkey agar containing the cefixime, tellurite supplement (CT-SMAC; Oxoid, Hampshire, UK) and incubated for 18 h at 37°C. A total of 10 presumptive* E. coli* O157 colonies per sample were serotyped using an* E. coli* O157 Latex Test Kit (Oxoid, Hampshire, UK). All isolates agglutinating with the O157 antiserum were further characterized by polymerase chain reaction (PCR) to detect the presence of* rfbE, stx*_1_ *, stx*_2_ *, eaeA, ehxA*, and* fliC* genes using primers and reaction conditions as previously described [17].

Characterization of lineage-specific polymorphisms-6 (LSPA-6) of* E. coli* O157 isolates was performed using target amplification and capillary electrophoresis as described previously [18, 19]. An Applied Biosystems 3130 Genetic Analyzer (Applied Biosystems, California, USA) with a DS-33 matrix and GeneScan 600 LIZsize standard was used for capillary electrophoresis, while a Peak Scanner software (Version 1.0; Applied Biosystems, California, USA) was used to interpret amplicon sizes. LSPA-6 alleles were defined according to [18]. Isolates with LSPA-6 genotype 111111 or 211111 were classified as lineage I (LI) or lineage I/II (LI/II), respectively, while all other allele combinations were grouped as lineage II (LII) [18, 20].

Analysis of Shiga toxin encoding bacteriophage insertion sites (SBI) of* E. coli* O157 isolates was determined as previously described [21].

### 2.3. Detection, Isolation, and Characterization of Non-O157* E. coli*

Samples (10 g) which were initially stored at −70°C in TSB with 25% glycerol were diluted 1/10 in BPW, homogenized for 30 s, and incubated for 18 h at 37°C without agitation. DNA was extracted from 1 mL of the enriched sample using the Nucleospin Soli DNA extraction kit (Macherey Nagel, Düren, Germany) following the manufacturer's instructions. A multiplex PCR was used to screen enrichments for the presence of STEC virulence genes* stx*_1_ *, stx*_2_ *, eaeA*, and* ehxA* using primers and reaction conditions as described by A. W. Paton and J. C. Paton [22] with several modifications. A reaction volume of 25 *μ*L was used with 2 *μ*L of DNA template and final concentration of 0.25 *μ*M of each primer, 5x Green GoTaq Flexi Buffer (Promega, Madison, USA), 200 *μ*M of dNTP, 2 mM of MgCl_2_, and 1 unit of GoTaq DNA polymerase (Promega, Madison, USA). The PCR products were separated by electrophoresis on a 2% agarose gel, stained with ethidium bromide (0.5 *μ*g/mL) and visualized under UV light. Enriched samples positive for* stx* and* eaeA* by PCR were streaked on chromocult-TBX agar (Merck, Darmstadt, Germany) and coliformen agar enhanced selectivity (Merck, Darmstadt, Germany) and incubated overnight at 37°C. Following incubation, up to 50* E. coli* colonies per sample were chosen based on colony morphology and screened individually by multiplex PCR for the presence of* stx*_1_ *, stx*_2_ *, eaeA*, and* ehxA* as described above. Colonies that were positive for* stx* and* eaeA* were then tested for the “big 6”* E. coli* non-O157 serogroups by PCR using primers and conditions described previously [17, 23].

The enriched samples were also tested for the presence of genes specific to the “big 6”* E. coli* non-O157 serogroups. Samples that tested positive by PCR for any of the target serogroups were subjected to IMS for O26, O111, O103, and O145 using Dynabeads (Dynal, Oslo, Norway) following the manufacturer's instructions. The bead-bacteria complexes formed during IMS of O26 were plated onto rhamnose MacConkey agar, while those of O111, O103, and O145 were plated onto chromocult-TBX agar and coliformen agar-enhanced selectivity and incubated overnight at 37°C. Following incubation, 10 presumptive colonies (per sample) based on colony morphology were subjected to serogroup specific PCR and those confirmed as a specific serogroup were tested by PCR for the presence of STEC virulence genes. Isolation of serogroups O45 and O121 was performed on enriched fecal samples positive for STEC virulence markers which were directly plated onto chromocult-TBX agar as described above.

### 2.4. Biochemical Confirmation of* E. coli* Isolates

All the isolates were biochemically identified as* E. coli* by citrate utilization and indole production tests [24].

### 2.5. Bacterial Strains

The bacterial strains used as controls in this study are listed in Table 2.

### 2.6. Pulsed-Field Gel Electrophoresis (PFGE)

PFGE using* Xba*I was performed on all* E. coli* O157 and non-O157 isolates in a CHEF Mapper (Bio-Rad, California, USA) according to the standardized PulseNet protocol [25]. Banding patterns were analysed using BioNumerics software, version 6.5 (Applied Maths BVBA, Sint-Martens-Latem, Belgium) following the PulseNet protocol.

### 2.7. Subtyping of* stx* and Intimin (*eaeA*) Genes of* E. coli* O157 and Non-O157

The subtypes of* stx* and* eaeA* in isolates carrying these markers were determined following previously published methods [26, 27].

### 2.8. Detection of Shiga Toxin Expression

Stx expression by the STEC strains was determined according to the method adapted from Shringi et al. [28] using an ELISA kit (Premier EHEC, Meridian Bioscience, Ohio, USA). Mitomycin C (Sigma Aldrich, Missouri, USA) was used at a final concentration of 0.5 *μ*g/mL to induce Stx production. After induction, the cells were lysed using Polymixin B (Sigma Aldrich, Missouri, USA) at a final concentration of 0.5 mg/mL and incubated at 37°C for 1 h with rotary shaking (250 rpm). Polymixin B treated cultures were diluted 1 : 100 in sterile LB broth immediately followed by 1 : 2 dilution in sample diluent of the ELISA kit. Absorbance readings were obtained at wavelengths 450 nm and 630 nm using a Victor X microtiter plate reader (Perkin Elmer, Glen Waverley, Australia) and the results were displayed as the mean value of two independent biological replicates.

## 3. Results

### 3.1. Presence of STEC O157 and Virulence Factors

STEC O157 was isolated from 6 (4%) cattle feces samples, all of which were from farm A (Table 3). A total of 32 STEC O157 isolates were obtained from 6 different cattle feces samples. The isolates obtained were clustered into two different PFGE groups (at >92% similarity) with the majority of isolates (28 isolates from 5 different fecal samples) belonging to one PFGE group and the remaining isolates (4 isolates from a single fecal sample) belonging to the other. All 32 STEC O157 isolates were positive for the virulence factors* stx*_2_ *, eaeA*, and* ehxA* and also for* fliC* specific for the H7 antigen indicating they belong to the O157:H7 genotype. All samples from farms B–F were negative for the presence STEC O157.

LSPA-6 target amplification indicated that all the STEC O157:H7 isolates collected from cattle feces samples in farm A belong to lineage II (Table 3). According to the SBI genotyping code, genotype SY2c was observed in all STEC O157:H7 isolates collected from cattle feces samples in farm A indicating the association of *stx*_2c_ with prophage insertion in the* sbcB* locus (Table 3).

In addition, all STEC O157:H7 isolates obtained from UPM carried the virulence markers* stx*_1_ *, stx*_2_ *, eaeA*, and* ehxA* and belonged to a single PFGE group (at >92% similarity). They were of lineage I and contained the SBI genotype WY12 indicating the association of* stx*_1_ and *stx*_2a_ with prophage insertion in the* yehV* and* wrbA* loci, respectively.

### 3.2. Presence of Non-O157 STEC and Virulence Factors

In the initial PCR screen of the enriched samples, various combinations of virulence markers and genes for the target non-O157 serogroups were observed in all the farms except in farm F. Although samples in farm F were positive for different combinations of virulence markers, none of the samples were positive for any of the target non-O157 serogroups tested (Table 4). Overall, the combination of* stx* (either* stx*_1_ *, stx*_2_ or both) and* eaeA* was present in 32.3% (44 samples out of 136 samples), while the gene indicating the presence of serogroup O103 seemed to be predominant (44.1% of samples) among all 136 samples.

Although the initial PCR screening of the enriched feces samples indicated a relatively high number of samples with the target genes for the virulence factors and non-O157 serogroups, only 2 samples (1.5%) yielded non-O157 STEC isolates (Table 3). Both of these were cattle feces samples collected from farm A, from which two non-O157 STEC strains (negative for any of the “big 6” non-O157 serogroups) were isolated which belonged to two unique PFGE groups. One of the two non-O157 STEC isolates was positive for* stx*_1_ *, stx*_2_, and* ehxA* while the other isolate was positive for* stx*_1_ alone.

### 3.3. Characterization of* E. coli* Serogroups Lacking* stx*

*E. coli* of the target serogroups (O157 and the “big 6” non-O157) lacking* stx* but carrying other combinations of virulence markers were also isolated from ruminant feces samples (Table 5). These included* E. coli* of serogroups O157, O103, and O26 which were negative for any of the virulence markers, O157 which carried* eaeA* alone and O26 with* eaeA* and* ehxA*.

### 3.4. *stx* and* eaeA* Subtyping

All the STEC O157:H7 isolates collected from cattle feces samples in farm A were positive for *stx*_2c_ (Table 3), while all STEC O157:H7 isolates from UPM were positive for *stx*_1a_ and *stx*_2a_. One of the non-O157 STEC isolates was positive for *stx*_1a_, *stx*_2a_, and *stx*_2c_, while the other isolate was positive for *stx*_1a_ alone (Table 3). Overall, *stx*_2c_ was the more prevalent genotype among the* stx*_2_ positive isolates.

Two different* eaeA* subtypes out of the seven* eaeA* variants (*α*1, *α*2, *β*1, *β*2, *γ*1, *γ*2/*θ*, and *ε*) tested were present among the* eaeA* positive isolates of* E. coli* O157 and non-O157. The STEC O157:H7 isolates were positive for* eaeA*-*γ*1 (Table 3) while the two O26 isolates lacking* stx* were positive for* eaeA*-*β*1 (Table 5).

### 3.5. Shiga Toxin Production

Stx production of all the *stx*_2c_ positive STEC O157:H7 isolates collected from the cattle feces samples in farm A were below the level of detection. In contrast, all the *stx*_1a_ and *stx*_2a_ positive STEC O157:H7 isolates obtained from UPM produced a high amount of Stx similar to the positive control STEC O157:H7 isolates, ATCC 43895, EC543a, and EC6a. Of the two non-O157 STEC isolates, Stx production of the *stx*_1a_ positive non-O157 isolate was also below the level of detection. However, the *stx*_1a_, *stx*_2a_, and *stx*_2c_ positive non-O157 STEC isolate indicated a moderate amount of Stx production although lower than that observed for the UPM STEC O157:H7 isolates.

## 4. Discussion

In Malaysia, no studies have been conducted so far to characterize STEC O157 or non-O157 in ruminant feces. Thus, the goal of the present study was to gain insight on the virulence determinants of STEC O157 and non-O157 present in ruminant feces in Malaysia.

In this study, STEC O157 was isolated from 6 (4.4%) ruminant feces samples and non-O157 STEC was isolated from 2 (1.5%) of the ruminant feces samples. Several other authors have also reported low isolation rates (less than 10%) of STEC O157 and non-O157 in ruminant feces in tropical countries [8, 12]. However, this study was not adequate to determine the prevalence of STEC O157 and non-O157 in Malaysia and, thus, to obtain more comprehensive data on the prevalence of STEC O157 and non-O157 serogroups in Malaysia, sampling of a wider geographical area within Malaysia should be undertaken.

*E. coli* O157 populations have been shown to vary in their distribution among bovine and clinical sources due to their genotypic differences [29]. LSPA-6 analysis, a simple multiplex PCR assay, categorizes* E. coli* O157 strains into three different genotypes referred to as lineage I, lineage I/II, and lineage II. Isolates of lineage I and I/II are mostly associated with human clinical sources while lineage II isolates are mostly associated with bovine sources [18]. In this study, all the STEC O157:H7 isolates belonged to lineage II in contrast to STEC O157 isolates from countries such as Australia and USA where lineage I/II and lineage I predominates [30]. Interestingly, all the STEC O157:H7 isolates from UPM were of lineage I indicating the presence of STEC O157 isolates of both lineage I and II in bovine sources in Malaysia. STEC O157 isolates of lineage II are shown to be less virulent and possibly impaired in their transmissibility to humans compared to lineage I or I/II [31]. The presence of STEC O157 isolates of lineage II in ruminants in Malaysia from this study suggests that these isolates could have less pathogenic potential in humans.

Pathogenic potential of STEC isolates has also been shown to be associated with the presence of particular* stx* genotypes.* E. coli* isolates carrying* stx*_1_ or *stx*_2c_ are associated with low virulence potential compared to those which carry* stx*_2_ (*stx*_2a_) [32]. In this study, all the STEC O157:H7 isolates obtained carried *stx*_2c_ indicating low virulence potential in humans compared to the STEC O157:H7 isolates from UPM with *stx*_1a_ and *stx*_2a_. One of the two non-O157 STEC isolates of unknown serogroup with *stx*_1a_, *stx*_2a_, and *stx*_2c_ indicated a high pathogenic potential compared to the other isolate with *stx*_1a_ alone.

Not all* E. coli* isolates carrying* stx* produce Stx [33]. This was true for all *stx*_2c_ positive STEC O157:H7 isolates and one of the non-O157 isolates positive for *stx*_1a_ obtained in this study. In contrast, the UPM STEC O157 isolates produced Stx. Although the exact reasons for the discrepancy observed in Stx production of* stx* positive* E. coli* isolates from this study is not fully understood, previous studies have also identified* E. coli* isolates positive for* stx* but negative for Stx production [33, 34]. In fact, the study by Koitabashi et al. [34] suggested that* stx*_2_ positive* E. coli* O157 strains that produce little or no Stx2 may be widely distributed in the Asian environment.

Particular* stx* genotypes of STEC O157 have been shown to be associated both with particular SBI genotypes and with their relative frequency of isolation from clinical and bovine sources [21]. Clinical isolates are generally characterized by the carrying of* stx*_2_ and* stx*_2_-associated bacteriophage sequences adjacent to either* wrbA* or* argW* (SBI genotypes: WY12, AY2, ASY2, ASY22c), while bovine isolates are characterized by carrying of *stx*_2c_ and *stx*_2c_-associated bacteriophage sequences adjacent to* sbcB* (SBI genotypes: SY2c, SY12c, and ASY12c). In agreement with these observations, the STEC O157 isolates obtained from cattle feces from this study carried *stx*_2c_ with an occupied* sbcB* locus (SY2c). However, the STEC O157 from UPM which were collected from bovine sources carried* stx*_2_ and an occupied* wrbA *locus indicating characteristics of clinical isolates.

All the STEC O157:H7 isolates in this study and the STEC O157:H7 isolates from UPM carried* eaeA-γ*1 as reported for* eaeA* positive* E. coli* O157 in previous studies [27, 35]. None of the* eaeA* positive non-STEC O157 could be subtyped using the primers for* eaeA* subtypes *α*1, *α*2, *β*1, *β*2, *γ*1, *γ*2/*θ*, and *ε*. It is possible that these isolates belonged to other intimin subtypes such as *δ*/*κ*, *ζ*, *η*, *ι*, *λ*, *μ*, and *ν* which were not tested for in this study. The two* eaeA* positive* E. coli* O26 isolates carried* eaeA-β*1 similar to several other* E. coli* O26 isolates previously associated with human STEC strains that cause HUS [27].

## 5. Conclusions

Despite the use of specific and sensitive methods of enrichment and IMS followed in this study to isolate STEC O157 and non-O157, it appears that the presence of both STEC O157 and non-O157 in ruminant feces was low (4% and 1.5%, resp.). The *stx*_2c_ carrying STEC O157:H7 isolates of lineage II from this study suggests that these bacteria potentially represent a less pathogenic clone of STEC O157 in Malaysia. This together with the presence of STEC O157 and non-O157 in a small percentage of ruminants in this study could contribute to the reasons for the lack of reported sporadic cases and outbreaks caused by STEC O157 in Malaysia. Similar to STEC O157, the low percentage of non-O157 STEC isolates observed together with their low pathogenic potential indicated by the lack of* eaeA* and moderate to no Stx production suggests a low probability of causing disease in humans.

## Figures and Tables

**Table 1:** Distribution of ruminant feces samples collected from farms A–F.

Farm	Location	Ruminant feces samples				Total samples
		Cattle	Buffalo	Goat	Sheep	
A	Serdang	25	—	—	—	25
B	Kluang	9	20	7	8	44
C	Sentul	9	—	—	—	9
D	Gemas	24	—	—	—	24
E	Puchong	13	—	5	—	18
F	Lumut	16	—	—	—	16

**Table 2:** Bacterial strains used in the study.

Strain ID	Serogroup	Source	Country	Virulence traits
Sakai	O157	Radish sprouts	Japan	*stx*_1_, *stx*_2_ *, eaeA, ehxA *
ATCC 43895	O157	Ground beef	USA	*stx*_1_, *stx*_2_ *, eaeA, ehxA *
EC543a	O157	Cattle feces	Australia	*stx*_1_, *stx*_2_ *, eaeA, ehxA *
EC6a	O157	Cattle feces	Australia	*stx*_2_ *, eaeA, ehxA *
1 UPM^a^	O157	Bovine milk	Malaysia	*stx*_1_, *stx*_2_ *, eaeA, ehxA *
2 UPM^a^	O157	Bovine milk	Malaysia	*stx*_1_, *stx*_2_ *, eaeA, ehxA *
3 UPM^a^	O157	Beef	Malaysia	*stx*_1_, *stx*_2_ *, eaeA, ehxA *
4 UPM^a^	O157	Beef	Malaysia	*stx*_1_, *stx*_2_ *, eaeA, ehxA *
MG1655 (*E. coli* K-12)	OR:H48:K-^b^	Laboratory strain	USA	None
EC3008a^c^	O26	Cattle feces	Australia	*eaeA *
EC3009a^c^	O45	Cattle feces	Australia	None
EC2998a^c^	O103	Cattle feces	Australia	None
EC3113a^c^	O111	Cattle feces	Australia	None
EC3111a^c^	O121	Cattle feces	Australia	None

^a^Provided by Professor Son Radu at Universiti Putra Malaysia.

^b^OR = O antigen rough strain which does not produce a typeable O antigen.

^c^ *E. coli* non-O157 strains used as controls in the study, provided by Lesley Duffy at CSIRO, Brisbane, Australia.

**Table 3:** STEC O157 and non-O157 and their virulence profiles.

STEC serogroup	Number of STEC+ samples (%)	Source	Number of isolates	Virulence factors	Lineage	SBI profile
O157:H7^a^	6 (4%)	Cattle feces	28^b^	*stx*_2c_, *eaeA-γ*1, *ehxA *	II	SY2c
			4^b^	*stx*_2c_, * eaeA-γ*1 *ehxA *	II	SY2c

Non-O157^a^ (unknown)	2 (1.5%)	Cattle feces	1	*stx*_1a_, *stx*_2a_, *stx*_2c_, *ehxA *	—	—
			1	*stx*_1_	—	—

^a^Isolates of STEC O157:H7 and non-O157 were only present in samples obtained from farm A. All samples from farms B–F were negative for STEC O157:H7 and non-O157 isolates.

^b^On farm A, 28 of the STEC O157 isolates belonged to one PFGE group (at >92% similarity) and the remaining 4 isolates belonged to another PFGE group.

—: not applicable.

**Table 4:** Occurrence of target virulence factors and “big 6” non-O157 serogroups in the initial PCR screen of the enriched samples from each farm (A–F).

Farm	Number of samples tested	Percent positive for virulence gene combinations^a^								Percent positive for serogroups^a^					
		*stx*_1_, *stx*_2_ *, eaeA, ehxA *	*stx*_1_, *stx*_2_ *, ehxA *	*stx*_1_ or *stx*_2_ *, eaeA, ehxA *	*stx*_1_ *, ehxA *	*stx*_2_ *, ehxA *	*eaeA, ehxA *	*eaeA *	*stx*_1_ or *stx*_2_ alone	O111	O26	O121	O145	O45	O103
A	25	6.6	2.9	0	0	1.5	0	0	0	0	0	5.9	0	3.7	5.1
B	44	13.2	2.9	0	0	0	0	0	0	0.7	9.6	13.2	0	2.2	16.2
C	9	2.9	0	0	0	0.7	2.2	0.7	0	0	2.9	0.7	1.5	3.7	5.1
D	24	1.5	4.4	1.5	0	0	0.7	0	2.9	0	8.1	0	0.7	0.7	10.3
E	18	5.9	2.9	0.7	0	0	0	0	0	3.7	4.4	0.7	0.7	8.1	7.4
F	16	0	2.2	0	1.5	0	0	0	0	0	0	0	0	0	0

Total	136	30.1	15.4	2.2	1.5	2.2	2.9	0.7	2.9	4.4	25	20.6	2.9	18.4	44.1

^a^The percentage of samples positive were calculated by dividing the number of positive samples for each category in the initial PCR screen by the total number of samples (*n* = 136) collected.

**Table 5:** Isolation and virulence profiles of *E. coli* O157 and “big 6” *E. coli* non-O157 serogroups lacking *stx*.

Serogroup	Farm	Source	Number of + samples	Number of isolates tested	Virulence factors	Intimin subtype
O103	A	Cattle feces	3	3	None	—

O157	B	Cattle feces	1	2	*eaeA *	NT^a^
		Sheep feces	2	3	*eaeA* (1 isolate) none (2 isolates)	NT
		Buffalo feces	3	5	*eaeA *	NT

O26	B	Buffalo feces	2	5	*eaeA*, *ehxA* (2 isolates) none (3 isolates)	*eaeA-β1 *

O103	B	Buffalo feces	1	7	None	—

O26	C	Cattle feces	2	11	None	—

^a^NT = non-typable.

—: not applicable.
